# Improving nitrogen use efficiency of *japonica* rice by molecular marker-assisted selection of the *NRT1.1B* gene

**DOI:** 10.3389/fpls.2026.1786744

**Published:** 2026-08-17

**Authors:** Gang Li, Zhipeng Zhang, Chen Zhao, Jie You, Zhangrong Wen, Weiqin Jiang, Jian Wang, Baoshan Cheng, Weijun Xu, Di Wang, Hao Gao, Guolian Wang

**Affiliations:** Huaiyin Institute of Agricultural Science in Xuhuai Region of Jiangsu, Huai’an, Jiangsu, China

**Keywords:** germplasm resources, *Japonica* rice, molecular marker-assisted selection, nitrogen use efficiency, NRT1.1B gene

## Abstract

**Purpose:**

The gene *NRT1.1B* enhances nitrogen use efficiency (NUE) in rice. The aim of this study was to improve the NUE of the major *japonica* variety Huaidao 5 by introgressing the *indica NRT1.1B* allele using molecular marker-assisted selection.

**Methods:**

A functional marker, *NRT1.1B-KASP*, was developed from *indica*-*japonica NRT1.1B* variations and validated using F_2_ populations from 22 crosses. It introgressed the beneficial allele from donor AR56 into Huaidao 5. Nitrate uptake capacity and chlorate sensitivity were assessed, and the expression of key nitrogen metabolism genes was analyzed. Chlorate resistance assays were conducted to evaluate the NUE of Huaidao 5 and the improved line Xinhuai 5, measuring seedling growth inhibition. Field trials at multiple nitrogen levels (0–330 kg N hm^-^²) were used to analyze agronomic traits, yield, and various NUE parameters. Rice quality and blast resistance were assessed according to national industry standards.

**Results:**

Xinhuai 5 exhibited significantly enhanced nitrogen absorption and utilization, with increases in nitrogen accumulation, harvest index, agronomic efficiency, physiological efficiency, and partial factor productivity. Increased shoot ¹^5^N accumulation, great chlorate sensitivity, and upregulated expression of nitrate transporter and assimilation genes were also observed in Xinhuai 5. Its yield under 30% nitrogen reduction matched that of Huaidao 5 under normal conditions. Agronomic traits, including more effective tillers, higher seed setting rate, shortened growth period, and reduced plant height, improved. Furthermore, Xinhuai 5 demonstrated superior eating quality and enhanced blast resistance than Huaidao 5, with no significant difference in processing or appearance quality.

**Conclusion:**

The developed *NRT1.1B-KASP* marker enabled efficient introgression of the *NRT1.1B* gene into *japonica* rice, significantly improving the NUE. Our results provide a scientific basis and germplasm resources for improving NUE of *japonica* rice.

## 1 Introduction

Rice (*Oryza sativa* L.) is one of the most important food crops in the world and a staple food for more than half of the world’s population. China is the world’s largest rice producer and consumer (Song et al., 2022). However, the rice cultivation in China faces severe challenges due to excessive nitrogen fertilizer application. In 2021, total fertilizer use in China reached 17.453 million tons, accounting for 37% of global rice nitrogen consumption, despite China having only 20% of the world’s rice planting area. This overreliance on nitrogen fertilizers has led to soil degradation and extremely low nitrogen use efficiency (NUE), serious waste, groundwater pollution, decline in the quality of agricultural products, and low efficiency of agricultural planting (Meng et al., 2017). There is an urgent need to develop high-efficiency, eco-friendly rice varieties and sustainable cultivation practices.

At present, the average nitrogen application rate of rice in China is 209 kg/hm^2^, which is 90% higher than the world average (Mekouar, 2018), with regions like Jiangsu exceeding 300 kg/hm^2^ (Wu et al., 2016; Gu et al., 2017). Excessive use of nitrogen fertilizer has led to 30-35% lower NUE in China than in other rice growing countries (Peng et al., 2010; Zhang et al., 2013). While unfertilized fields maintain higher agronomic efficiency due to reliance on soil nitrogen background, prolonged overfertilization has resulted in high residual soil nitrogen, further reducing efficiency and exacerbating environmental pollution such as water eutrophication (Peng et al., 2010). Therefore, improving the NUE of rice and strengthening the nutrient management of rice, which are of great significance to the sustainable development of agriculture and environmental protection, are urgent problems to be solved in rice production in China.

In recent years, Chinese scientists have cloned and identified several genes that are expected to improve rice NUE and increase yield. Several nitrogen-efficient genes, such as *NRT1.1A* (Wang et al., 2020), *NRT1.1B* (Zhang et al., 2019; Wang et al., 2020), and *TCP19* (Liu et al., 2021), have been cloned by Dr. Chu’s team. Among them, the *NRT1.1B* gene led to the difference of NUE between the *indica* and *japonica* subspecies. After introducing the *NRT1.1B* gene of *indica* varieties into *japonica* rice varieties, the yield under the condition of halving nitrogen fertilizer increased by 30-33% and the NUE increased by 30% compared with that in the control. Under normal fertilization conditions, yield increased by 8-15%, and NUE increased by about 10% (Liu et al., 2018). Therefore, the *NRT1.1B* gene can be used as an important target gene locus for genetic improvement of nitrogen efficiency in *japonica* rice. However, the practical application of *NRT1.1B* in *japonica* breeding has been limited by the lack of efficient and high-throughput genotyping tools for precise selection.

Kompetitive allele specific PCR (KASP) technology is widely used in rice molecular marker-assisted breeding (Steele et al., 2018), due to its high-throughput capability and precision in biallelic discrimination of known single-nucleotide polymorphism (SNPs) and insertions and deletions (InDels) across diverse genomic DNA samples, offering substantial advantages for large-scale genotyping (Semagn et al., 2014). Therefore, we developed a robust KASP marker specifically targeting the functional SNP in *NRT1.1B*, and successfully applied it to breed an improved line of the *japonica* variety Huaidao 5. The improved line exhibited significantly enhanced NUE under reduced nitrogen input, demonstrating the successful integration of genetic improvement and cultivation practices. This study provides valuable germplasm and an efficient technical strategy for achieving sustainable rice production through nitrogen-efficient breeding.

## 2 Methods

### 2.1 Plant materials

The recipient parent used in this study was Huaidao 5, a high-yielding, high-quality, and late-maturing medium *japonica* rice variety developed by Huaiyin Institute of Agricultural Sciences in the Xuhuai area of Jiangsu Province. The average yield of a 2-year regional test was 2.6% higher than that of the control, and it was the rice variety with the longest promotion time and the largest area in Jiangsu Province (Hui et al., 2023). The donor parent was AR56, a nitrogen-efficient germplasm carrying the *NRT1.1B* gene. The genetic combination was derived from the cross: Xiushui 134/////Xiushui 134//Nipponbare//Nipponbare//Nipponbare/IR24. This material was provided by the Institute of Genetics and Developmental Biology, Chinese Academy of Sciences.

### 2.2 Determination of NUE of main varieties and sequence analysis of *NRT1.1B* gene

Three typical *japonica* rice varieties (Huai 119, Suxiu 867, and Nanjing 9108) and three typical *indica* rice varieties (Huanghuazhan, Yangdao 6, and Fengliangyou 4) were selected as experimental materials to elucidate the difference in NUE among rice varieties and to identify the key loci of the *NRT1.1B* gene in genomic DNA of *indica* and *japonica* rice varieties (**Supplementary Table 1**). Information on rice varieties can be found in the China Rice Data Center (http://www.ricedata.cn/variety/). Nitrogen isotope tracer analysis and sequencing of the *NRT1.1B* gene were subsequently performed.

Nitrogen absorption and utilization rates of the six varieties were measured using an enhanced nitrogen tracer method based on stable isotope ratio mass spectrometry with a Delta Plus XP instrument (Thermo Fisher Scientific, Waltham, MA, USA). The complete sequence of *NRT1.1B* was obtained from the Rice Annotation Project Database (https://rapdb.dna.naro.go.jp), and the specific primers were designed (**Supplementary Table 2**). Whole-genome DNA was used as template for PCR amplification and subsequent sequencing.

### 2.3 Design and synthesis of KASP primers for *NRT1.1B* gene

According to the specific SNP locus (C/T) in *NRT1.1B* that differentiated *indica* and *japonica* rice, the following KASP primers were designed: forward primer Primer_-_Allele sequence: 5′-CAGCCTCCACTTGCTCGCCA-3′; reverse primer Primer_-_AlleleY sequence: 5′-AGCCTCCACTTGCTCGCC-3′; universal primer Primer_-_Common sequence: 5′-GACAGGTCGGCGGCGGAGT-3′.

### 2.4 PCR amplification and detection

Genomic DNA of the control and the test sample was isolated using the CTAB method. PCR amplification was performed in a 5 μL reaction volume containing 2.5 μL template DNA, 2.5 μL of 2× KASP Master Mix, and 0.07 μL assay mix. The thermal cycling conditions were as follows: initial denaturation at 94 °C for 15 min; 10 cycles of denaturation at 94 °C for 20 s, annealing/extension for 60 s with a starting temperature of 61 °C decreasing by 0.6 °C per cycle; and 26 cycles of denaturation at 94 °C for 20 s and annealing/extension at 55 °C for 60 s. Genotyping was performed by detecting the fluorescence signals using KASP genotyping after PCR amplification. The expression levels of key genes involved in nitrogen metabolism were quantitatively analyzed with three replicates. Primer sequences of target genes are provided in **Supplementary Table 2**.

### 2.5 NUE analysis

Chlorate resistance has been established as a reliable indicator of the nitrogen absorption and utilization capacity of plants (Karunarathne et al., 2021). Chlorate-treated seed germination assays and potassium chlorate hydroponic experiments were conducted using Huaidao 5 and its improved lines to investigate the effect of *NRT1.1B* gene on nitrogen absorption and utilization efficiency of rice.

#### 2.5.1 Potassium chlorate soaking germination assay

Seeds of both wild-type varieties and improved lines were placed in Petri dishes lined with filter paper. A 0.02% potassium chlorate solution was added, and germination was carried out under 14/10 h light/dark photoperiod with a corresponding 30 °C/25 °C temperature cycle. Seeds treated with distilled water served as the control. The potassium chlorate solution was replenished every 2 days. The shoot length of the seedlings was measured after 7 days. The chlorate resistance (CR) value, which reflects the variation in nitrogen absorption and utilization efficiency, was calculated as follows (Hu et al., 2015):


$$CR\%\;=\;Average\;seedling\;length\;in\;KClO_{3}/Average\;seedling\;length\;in\;water\;\times \;100\%$$

#### 2.5.2 Potassium chlorate hydroponic culture experiment

Seedlings of Huaidao 5 and its improved lines were grown in Kimura B nutrient solution (1000× concentration, prepared from four stock solutions, pH 5.5-5.8) for 10 days. Then, 0.5 mmol L^-1^ potassium chlorate was added to the solution. The leaves were photographed after 7 days of treatment, and the pigment contents were measured. The Kimura B nutrient solution was replaced every 2 days throughout the experiment (Hu et al., 2015).

### 2.6 Rice quality evaluation and blast resistance identification

Rice quality was evaluated in accordance with the industry standard NY/T 593-2021 “Edible Rice Variety Quality” (Ministry of Agriculture of the People’s Republic of China, 2021). The analysis was performed by the Food Quality Supervision, Inspection and Testing Center (Wuhan) of the Ministry of Agriculture and Rural Affairs. Evaluation of rice blast resistance, including disease investigation and rating, was conducted in accordance with the industry standard NY/T 2646-2014 “Technical Regulations for the Identification and Evaluation of Rice Blast Resistance in Rice Variety Testing” (Ministry of Agriculture of the People’s Republic of China, 2014). The resistance identification was conducted by the Institute of Plant Protection, Jiangsu Academy of Agricultural Sciences. The comprehensive resistance index was calculated using the following formula:


Comprehensive index = Leaf blast grade × 25% + Spike blast incidence grade × 25% + Spike blast loss index disease grade × 50%

### 2.7 Nitrogen fertilizer application experiment on improved lines

In 2022, a field experiment with three nitrogen application levels (low, medium, and high) was conducted at the experimental station of Huaiyin Institute of Agricultural Sciences in the Xuhuai region of Jiangsu Province by using the *NRT1.1B* gene-improved line and its recurrent parent Huadao 5. The experiment comprised 18 plots with three replicates. Each plot contained 8 rows with 9 plants per row, totaling 72 plants, and covered an area of 3 m^2^ (**Supplementary Table 3**). Integrated pest management practices were applied throughout the growth period to minimize yield loss, and other field management procedures followed local standard. The initial heading date was recorded for each plot and converted into days from sowing to heading. The panicle number of 10 plants per plot was examined at maturity to calculate the number of panicles per plant. Five individual plants were sampled to measure key agronomic traits, including plant height (PH), spike length (SL), grain number per spike (GNK), fruiting rate (FR), and thousand-grain weight (TKW). The remaining plants were bulk-harvested, threshed, dried, and adjusted to a moisture content of 14.5% before yield measurement (Si et al., 2016).

Huaidao 5 and Xinhuai 5 were used as experimental materials in the experimental field of Yangzhou University Agricultural College in 2022, and four fertilizer treatments of 0,150, 240, and 330 kg N hm^-2^ were set up. Each treatment was repeated three times to determine the nitrogen accumulation, nitrogen harvest index, nitrogen uptake efficiency, nitrogen agronomic efficiency, nitrogen physiological efficiency, and nitrogen partial factor productivity of the *NRT1.1B* gene under a conventional rice background (Fei et al., 2023).

### 2.8 Field NUE test and adaptability identification

Field application experiments were conducted to investigate the adaptability of the Huaidao 5 improved line to direct seeding and mechanical transplanting methods in field production.

The normal fertilization treatment (303.75 kg N hm^-2^) and nitrogen reduction treatment (232.95 kg N hm^-2^) were set up with Huaidao 5 as the control. A base fertilizer of the same compound fertilizer was applied, with urea divided into two treatments: normal application and 70% application. All plots received 450 kg/hm^2^ of compound fertilizer at sowing (June 15). Tillering fertilizer was applied on July 13: high fertilizer treatment received 225 kg/hm^2^ urea, and nitrogen treatment received 157.50 kg/hm^2^ urea. Panicle fertilizer was applied on August 4: high fertilizer treatment received 300 kg/hm^2^ urea, and nitrogen treatment received 210 kg/hm^2^ urea.

### 2.9 ^15^N accumulation assay

The ^15^N accumulation capacity of Huaidao 5 and its improved lines was evaluated after labeling with ^15^N-nitrate and ^15^N-ammonium (Hu et al., 2015). Seedlings were pre-cultured for 10 days in a modified Kimura B nutrient solution containing 5 mM KNO_3_​ as the nitrogen source. Seedlings were then exposed to the same solution in which KNO_3_​ was replaced with ^15^N-labeled KNO_3_​ (98 atom% ^15^N-KNO3​, Sigma-Aldrich) for 24 hours. To remove residual isotope from the root surface, plants were sequentially rinsed with unlabeled solution for 3 min and 0.1 mM CaSO_4_​ for 2 min. After separation into roots and shoots, samples were dried at 70 °C, subsequently homogenized to a fine powder, and subjected to ^15^N content analysis using an isotope ratio mass spectrometer.

### 2.10 Chlorate-sensitivity assay

A chlorate-sensitivity assay was conducted to compare the nitrogen utilization capacity between the wild-type Huaidao 5 and its improved lines. Following germination, uniformly grown seedlings of both genotypes were cultured for 4 days in a modified Kimura B solution supplemented with 2 mM KNO_3_. Subsequently, the seedlings were transferred and maintained in a treatment solution containing 2 mM potassium chlorate for 4 days, followed by a 2-day recovery period in KNO_3_-containing Kimura B solution. Chlorate sensitivity was quantified as the percentage of growth inhibition and was calculated using the following formula:


(Plant height of control − Plant height of chlorate treatment)/Plant height of control × 100%

### 2.11 Statistical analysis

Data analysis was performed using Microsoft Excel 2019 (Microsoft, Redmond, USA). Descriptive statistics, including means and standard deviations (SD), were calculated for all quantitative variables. For comparisons between groups, statistical significance was evaluated with a predetermined threshold of *P*< 0.05, which was applied consistently across all analyses.

## 3 Results

### 3.1 Determination of NUE of main varieties and sequence analysis of *NRT1.1B* gene

**Figure 1:**
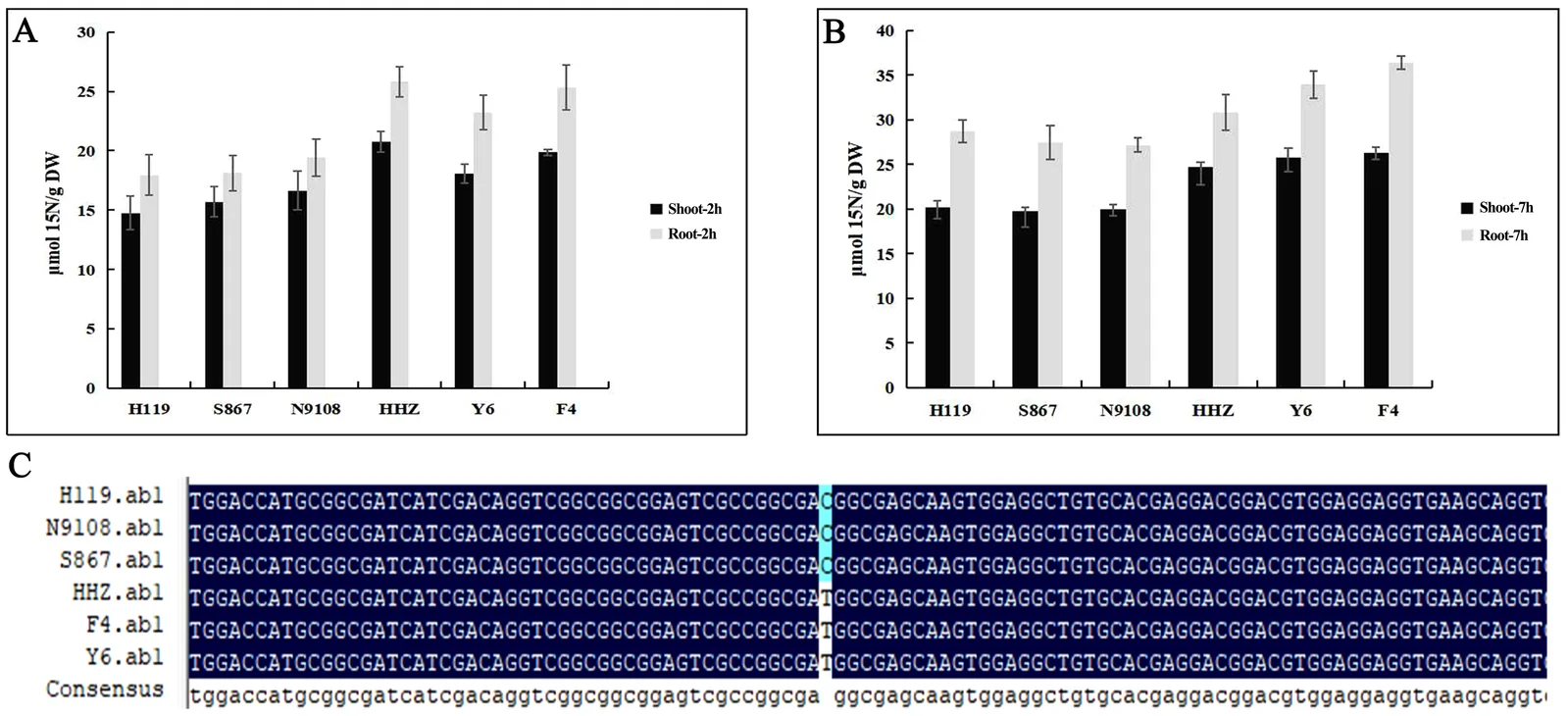
Determination of nitrogen absorption and utilization rate among varieties by stable isotope ratio mass spectrometry. **(A, B)** were shoot and root nitrogen uptake efficiency at 2 hours and 7 hours, respectively. **(C)** Sequencing analysis of the base differences between *japonica* and *indica* rice varieties at the key sites of *NRT1.1B* gene. H119: Huai119; S867: Suxiu 867; N9108: Nanjing 9108; HHZ: Huang huazhan; Y6: Yangdao 6; F4: Feng liangyou 4.

Nitrogen uptake and utilization efficiency were compared between three *japonica* (Huai 119, Suxiu 867, and Nanjing 9108) and three typical *indica* (Huanghuazhan, Yangdao 6, and Fengliangyou 4) rice varieties using stable isotope ratio mass spectrometry. *Indica* varieties exhibited significantly higher nitrogen uptake in both shoots and roots than the *japonica* varieties (**Figures 1A, B**).

Sequence analysis of the *NRT1.1B* gene revealed a C/T polymorphism at position 980 in the coding region of the *NRT1.1B* gene, leading to an amino acid substitution for *japonica* (Threonine) and *indica* rice (Methionine) (**Figure 1C**).

### 3.2 Development and validation of a KASP marker for *NRT1.1B*

**Figure 2:**
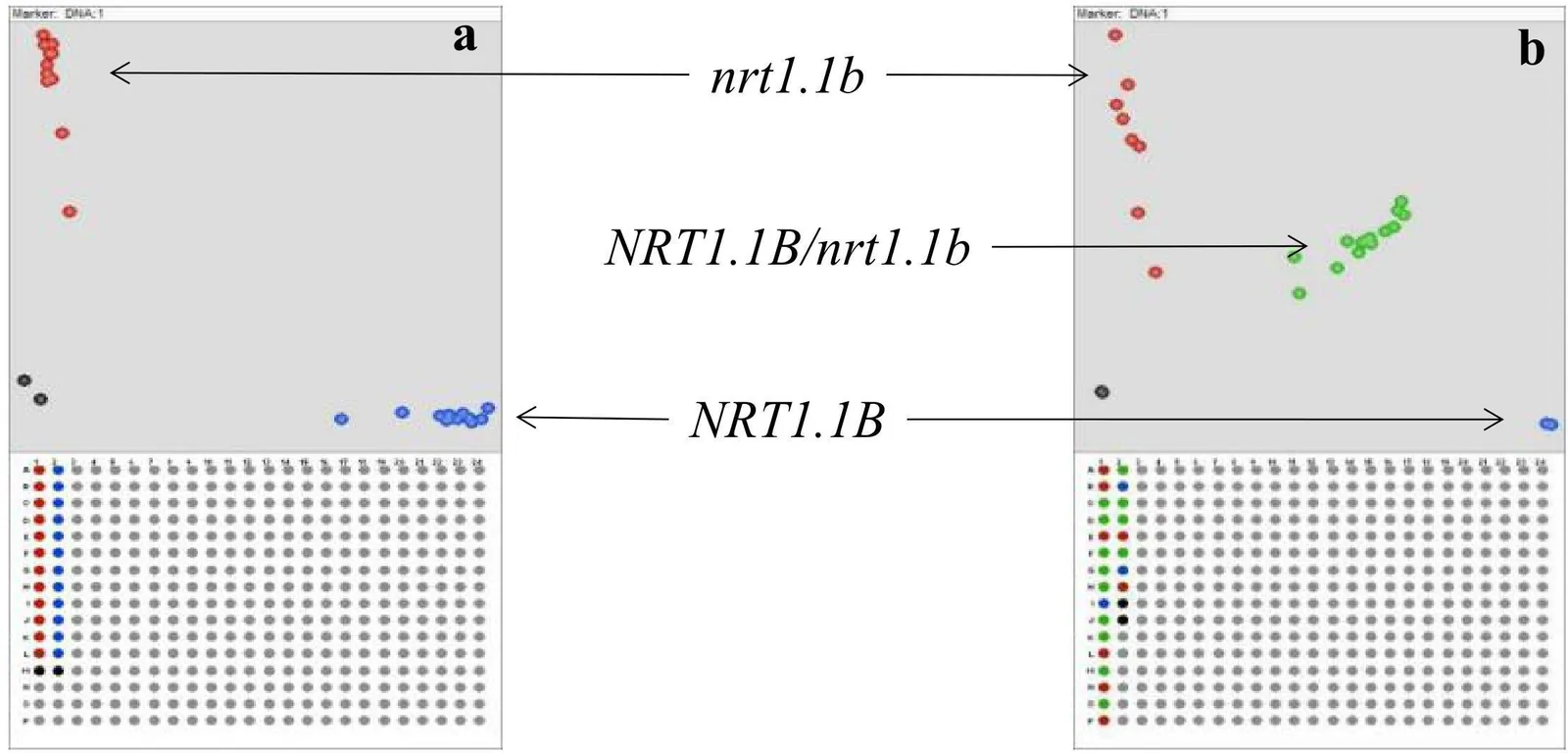
KASP typing results. **(A)** KASP marker genotyping results of 12 *indica* rice varieties and 12 *japonica* rice varieties; **(B)** KASP marker genotyping results of 22 F_2_ segregating populations and 2 parental genotypes. If the fluorescence of the sample product to be tested showed the same color as the positive control, the rice variety to be tested carried the *indica NRT1.1B*; on the contrary, the rice varieties to be tested contain *japonica nrt1.1b*. Black fluorescence indicates blank control.

Genotyping based on fluorescence signals showed that all 12 *indica* varieties exhibited blue fluorescence (indicating the presence of the *NRT1.1B* allele), while all 12 *japonica* varieties showed red fluorescence (homozygous for the *nrt1.1b* allele) (**Figure 2**). This confirmed clear differentiation between the two subspecies at the target locus. Furthermore, 22 *indica*-*japonica* rice F_2_ segregating populations were genotyped, with Yangdao 6 and Huadao 5 as positive and negative controls. The results showed that the 22 F_2_ populations were divided into three genotypic categories: 2 homozygous *NRT1.1B*, 7 homozygous *nrt1.1b*, and 13 heterozygous plants (**Figure 2**). The accuracy and reliability of the *NRT1.1B-KASP* marker, which could be used as a new large-scale functional gene detection marker for *NRT1.1B* genotyping, were verified.

### 3.3 Development of the *NRT1.1B* improved line of *japonica* rice by molecular marker-assisted selection

Huaidao 5 was crossed with the nitrogen-efficient donor AR56 (carrying the *NRT1.1B* gene) to generate F_1_ hybrids (designated as F128). The true hybrids were identified using KASP markers and subsequently backcrossed with Huaidao 5. Through six successive generations of backcrossing and marker-assisted selection, plants carrying the target allele were consistently identified using the *NRT1.1B-KASP* marker and recurrently backcrossed with Huaidao 5. BC_1_F_1_ individual plants were planted, screened using the *NRT1.1B-KASP* marker, and backcrossed with Huaidao 5. The selected plants from each backcross generation (BC_2_F_1,_ BC_3_F_1,_ BC_4_F_1,_ BC_5_F_1_) were used as the male parents in further backcrossing with the recurrent female parent Huidao 5.

**Figure 3:**
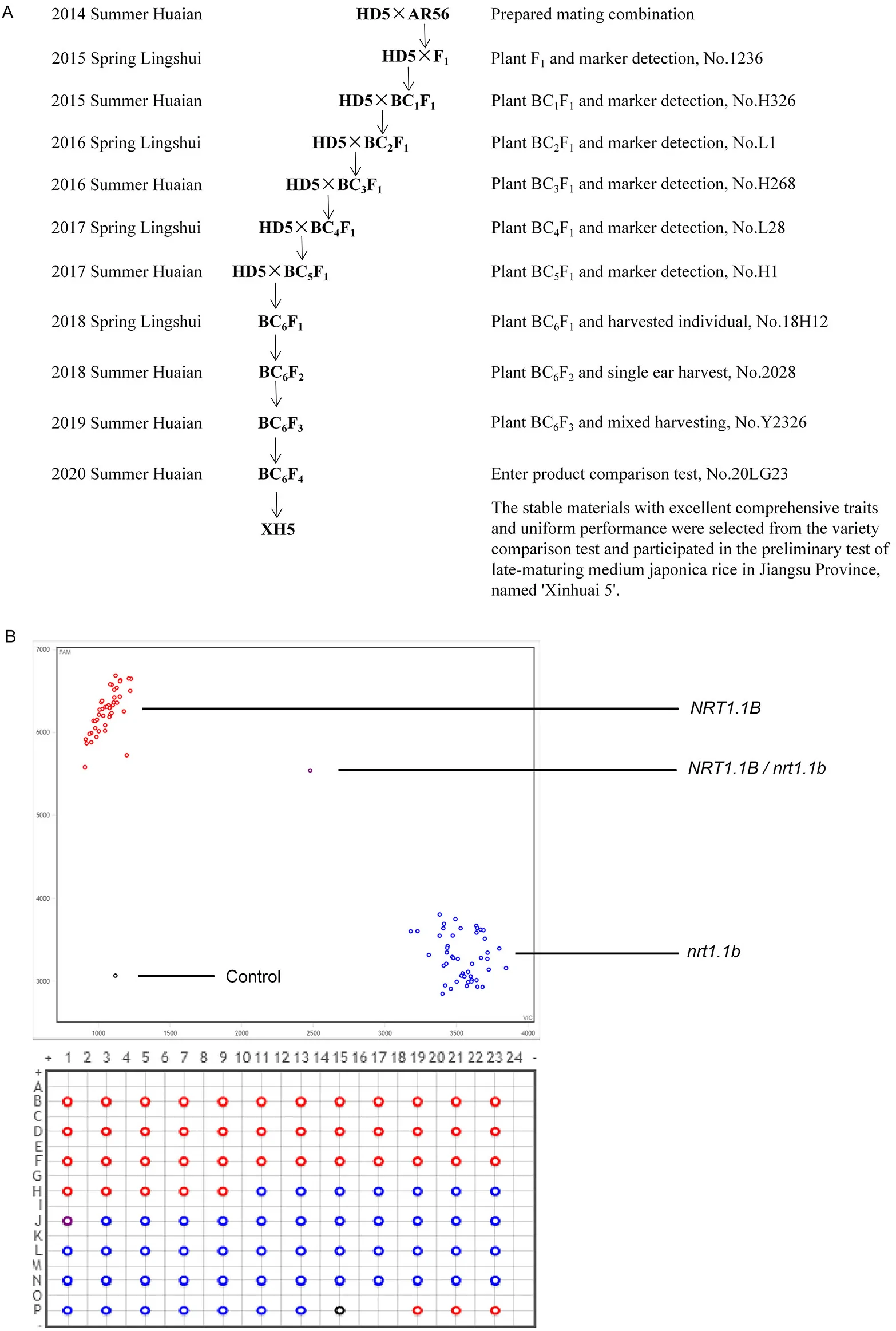
Molecular improvement of Xinhuai 5. **(A)**, the flow chart of molecular improvement process. **(B)**, genetyping validation of Xinhuai 5, compared with Huaidao 5.

Seeds were harvested from all BC_6_F_1_ generation plants. From the resulting BC_6_F_2_ population, plants homozygous for the *NRT1.1B* gene and exhibiting desirable agronomic traits were selected using marker detection. The BC_6_F_3_ generation population was planted, and the seeds were mixed after marker detection and removal of hybrid plants (**Figure 3A**). A total of 41 samples of Xinhuai 5 (the improved line) and 50 samples of Huaidao 5 (the control line) were genotyped at the *NRT1.1B* locus. As shown in **Figure 3B**, all Xinhuai 5 samples are present with *NRT1.1B* allele (red fluorescence), indicating *indica* NRT1.1B genotype. Among Huaidao 5 samples, one exhibited purple fluorescence *(indica-japonica* heterozygous), while the remaining displayed blue fluorescence, corresponding to the *japonica nrt1.1b* genotype. These suggested that the *KASP* marker-assisted selection process successfully introgressed the *indica NRT1.1B* allele into Huaidao 5, replacing its native *japonica nrt1.1b* allele to enhance NUE (**Figure 3B**).

### 3.4 Evaluation of the NUE of the *NRT1.1B* improved line

**Figure 4:**
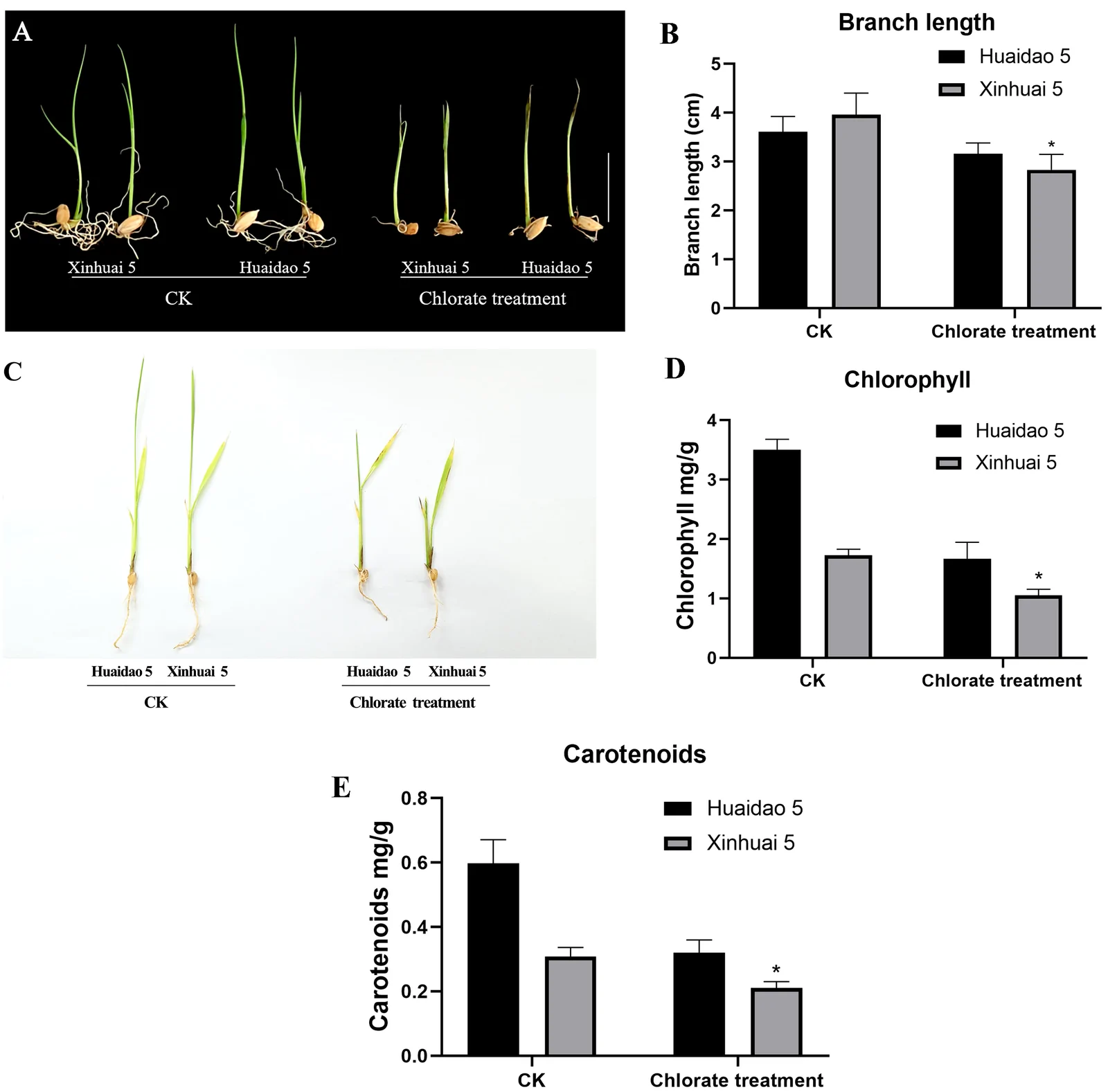
Resistance analysis of Xinhuai 5 and Huaidao 5 under chlorate treatment. **(A)** Chlorate-treated seedlings at germination stage, scale = 2 cm; **(B)** Branch length of chlorate-treated seedlings at germination stage, n = 10; **(C)** chlorate-treated seedlings at seedling stage, scale = 5 cm; **(D, E)** Total chlorophyll **(D)** and carotenoid **(E)** contents of seedlings after chlorate treatment at seedling stage. *P< 0.05.

The NUE of Xinhuai 5 was initially assessed using a chlorate resistance assay. The CR value of Huaidao 5 (84.79%) was significantly higher than that of Xinhuai 5 (77.26%), indicating greater chlorate uptake and enhanced nitrate absorption capacity in the improved line (**Supplementary Figure 1**). Although no significant difference in branch length was observed between the two lines under water-soaked conditions, Xinhuai 5 exhibited significantly reduced branch growth under potassium chlorate treatment (**Figures 4A, B**). The upper leaves of Xinhuai 5 withered after treatment, whereas the leaves of Huaidao 5 remained relatively less affected (**Figure 4C**). Furthermore, total chlorophyll and carotenoid contents were significantly lower in Xinhuai 5 than in Huaidao 5 under chlorate stress (**Figures 4D, E**). Quantitative analysis revealed that chlorate treatment reduced carotenoid content by 46.38% in Huaidao 5 (from 0.5974 ± 0.0746 to 0.3203 ± 0.0397 mg/g) and by 31.65% in Xinhuai 5 (from 0.3075 ± 0.0282 to 0.2102 ± 0.0202 mg/g). All these supported the higher chlorate uptake and nitrate absorption capacity of Xinhuai 5.

**Figure 5:**
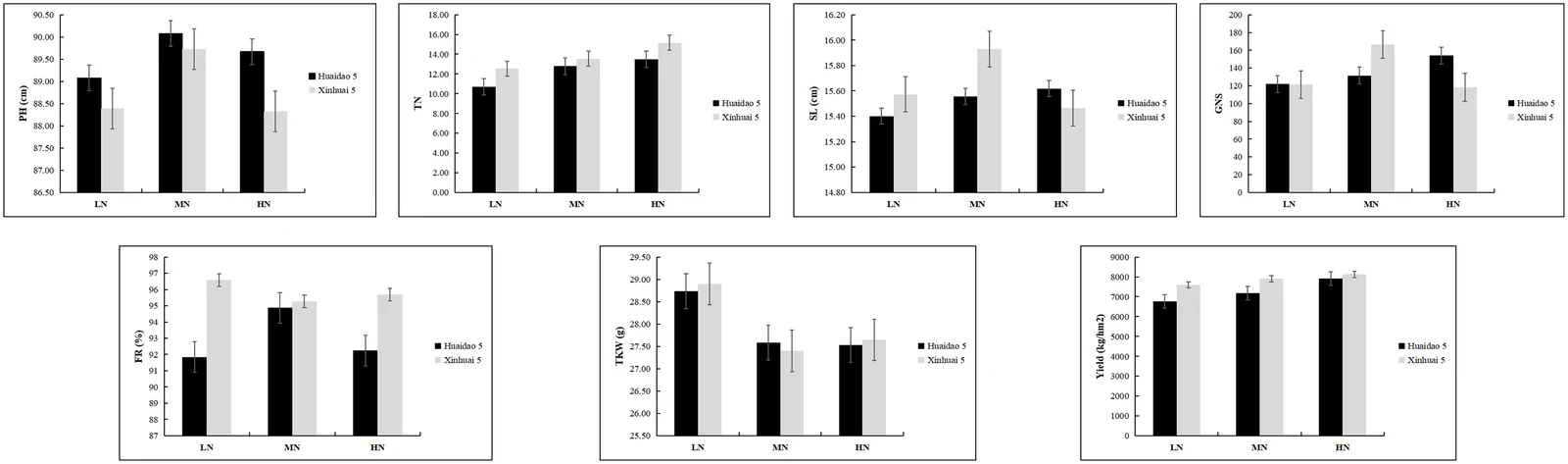
Yield and agronomic traits of *NRT1.1B* gene improved lines.

**Figure 6:**
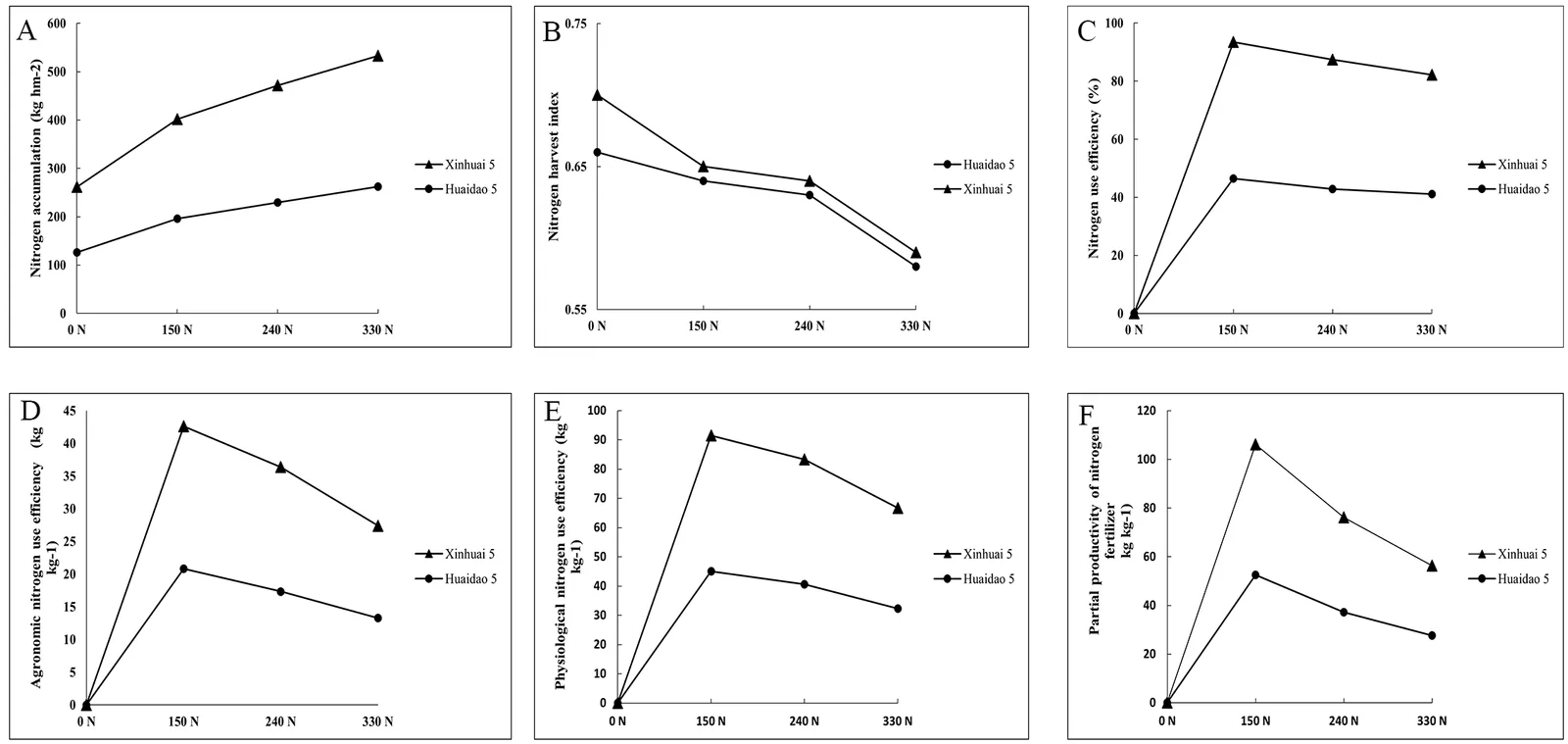
Nitrogen utilization indexes of Huaduo 5 and Xinhuai 5 under four fertilizer treatments; **(A)** Nitrogen accumulation; **(B)** Nitrogen harvest index; **(C)** Nitrogen use efficiency; **(D)** Agronomic nitrogen use efficiency; **(E)** Physiological nitrogen use efficiency; **(F)** Partial productivity of nitrogen fertilizer.

Xinhuai 5 consistently outperformed Huaidao 5 under field conditions with three nitrogen levels (low, medium, and high). As the nitrogen application rate increased, Huaidao 5 exhibited a rise in tiller number and grains per panicle, accompanied by a decrease in thousand kernel weight, ultimately leading to an overall increase in yield (**Table 1**; **Figure 5**). In comparison, Xinhuai 5 flowered 2 days earlier than Huaidao 5 across all nitrogen levels (**Table 1**; **Figure 5**). Compared to Huaidao 5, Xinhuai 5 showed reduced plant height, increased effective tillers, higher seed setting rate, and improved yield under all nitrogen regimes (**Table 1**). In a separate experiment with four nitrogen application rates, Xinhuai 5 demonstrated significantly greater nitrogen accumulation, nitrogen harvest index, nitrogen uptake efficiency, agronomic efficiency, physiological efficiency, and partial factor productivity than Huaidao 5 (**Figures 6A–F**). Although nitrogen accumulation increased with higher fertilizer input, nitrogen harvest index and use efficiency declined across both varieties. Xinhuai 5 exhibited a plant height reduction of 3–5 cm and headed 3 days earlier than Huaidao 5 under machine transplanting. The yield of Xinhuai 5 reached 9718.8 kg/hm^2^ under nitrogen reduction treatment, which was 7.03% higher than that of Huaidao 5 under the same treatment. Under normal nitrogen treatment, the yield of Xinhuai 5 was 9606.0 kg/hm^2^, which was 1.07% higher than that of Huaidao 5 (**Table 2**). The results showed that Xinhuai 5 had a higher NUE and a higher yield potential under lower nitrogen conditions.

**Table 1:** Determination of nitrogen absorption and utilization efficiency among varieties by stable isotope ratio mass spectrometry.

Treatment	Material	PH	TN	SL	GNS	FR	TKW	Yield
**LN**	HD5	89.08	10.69	15.40	121.99	91.85	28.74	6761.71
	XH5	88.39	12.55	15.57	121.33	96.58	28.90	7598.80
**MN**	HD5	90.08	12.78	15.56	131.54	94.87	27.58	7183.59
	XH5	89.72	13.56	15.93	166.45	95.27	27.40	7905.62
**HN**	HD5	89.67	13.49	15.62	154.06	92.24	27.53	7913.96
	XH5	88.32	15.16	15.46	118.25	95.69	27.64	8129.06

LN, low nitrogen; MN, medium nitrogen; HN, high nitrogen; HD5, huaidao 5; XH5, xinhuai 5; PH, plant height, cm; TN, tiller number; SL, spike length, cm; GNK, grain number per spike; FR, fertility rate; %; TKW, thousand kernel weight, g; Yield, kg/hm2.

**Table 2:** Yield measurement of Huaidao 5 and its improved line by machine transplanting.

Cultivars	Normal Fertility		Nitrogen Fertilizer Reduction	
	Yield	Increasing range	Yield	Increasing range
HD 5	9504.15	/	9080.7	/
XH 5	9606	1.07	9718.8	7.03

Normal Fertility, compound fertilizer 600 kg/hm2, urea 525 kg/hm2; Nitrogen Fertilizer Reduction, compound fertilizer 600 kg/hm2, urea 367.5 kg/hm2; Yield: kg/hm2, Increasing range: %.

### 3.5 The nitrate use and chlorate sensitivity in *NRT1.1B* improved line

**Figure 7:**
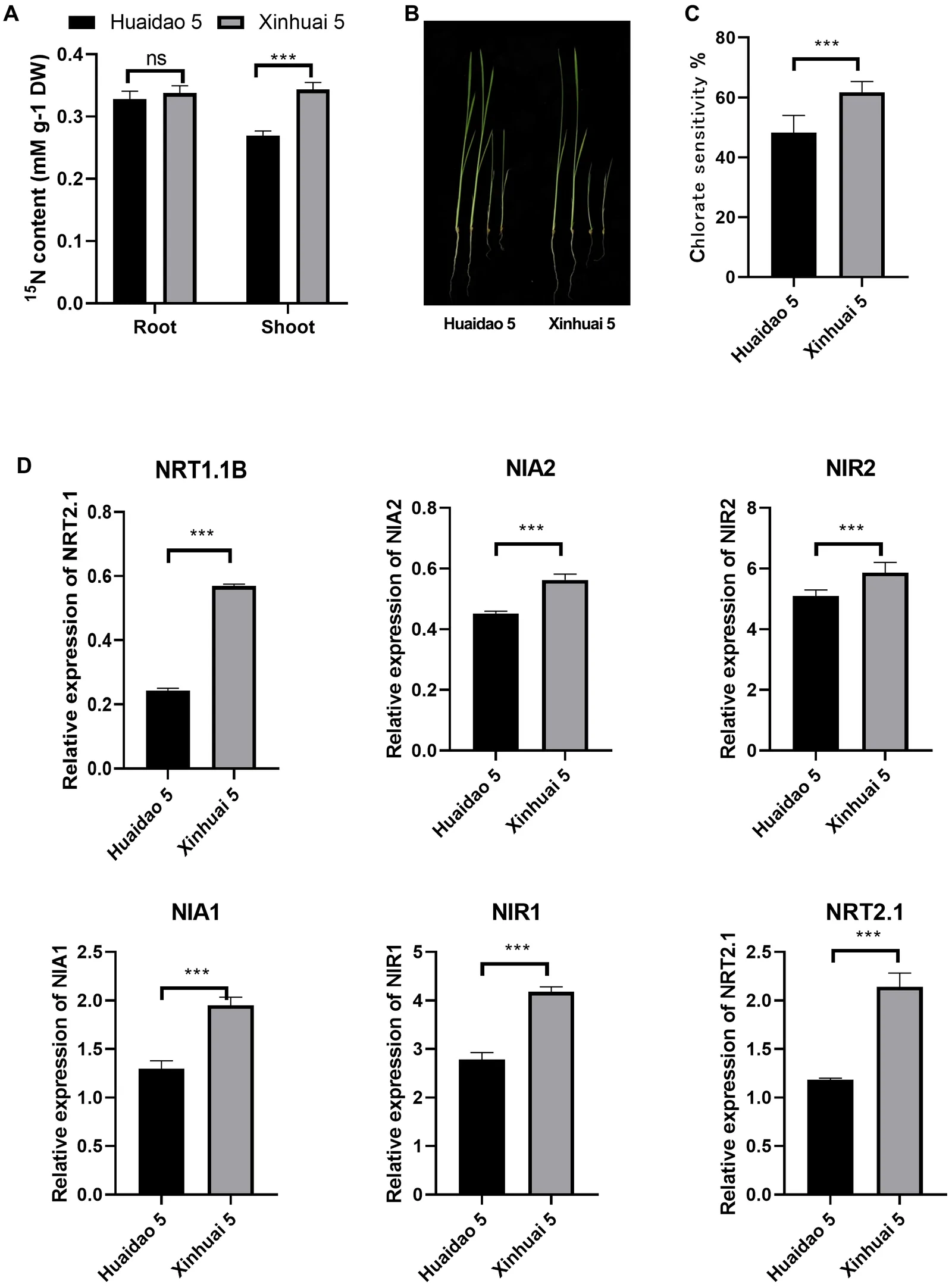
Nitrate use and chlorate sensitivity of Xinhuai 5 compared with Huaidao 5. **(A)** ¹^5^N accumulation in roots and shoots; **(B)** Huaidao5 and Xinhuai 5 after chlorate-sensitivity test. **(C)** Statistical analysis of chlorate sensitivity of Huaidao 5 and Xinhuai 5; **(D)** transcript expression of nitrogen metabolism related genes in roots of Huaidao 5 and Xinhuai 5. ***P<0.001; ns, not significant.

To evaluate nitrate uptake in *NRT1.1B* improved line, we performed ^15^N-accumulation assay in root and shoot of wild-type Huidao 5 and improved line Xinhuai 5. The results suggested that Xinhuai 5 and Huaidao 5 showed comparable levels of ¹^5^N accumulation in roots. Whereas, the ¹^5^N content in the shoots of Xinhuai 5 was significantly higher than that in Huaidao 5 (0.269 ± 0.008 mM g^-^¹ DW vs. 0.343 ± 0.011 mM g^-^¹ DW) (**Figure 7A**). The chlorate sensitivity assay revealed that the improved line Xinhuai 5 exhibited significantly higher chlorate sensitivity (61.62%) compared to the wild-type Huaidao 5 (48.22%) (**Figures 7B, C**). To elucidate the molecular mechanism underlying the improved NUE in the improved line, we analyzed the expression profiles of core nitrogen metabolism genes in the roots of Huaidao 5 and Xinhuai 5 under nitrate supply. As shown in **Figure 7D**, the transcript expression of the nitrate transporter genes *NRT1.1B* and *NRT2.1* were markedly higher in the improved line. Consistent with enhanced nitrate uptake, the expression of genes encoding nitrate reductase (*NIA1, NIA2*) and nitrite reductase (*NIR1, NIR2*), were also significantly elevated in Xinhuai 5. These demonstrated a superior capacity for nitrogen translocation and utilization in the improved line Xinhuai 5.

### 3.6 Quality evaluation and blast resistance identification of the *NRT1.1B* improved line

Quality analysis revealed that compared to Huaidao 5, Xinhuai 5 showed slight but non-significant decreases in milled rice rate, head milled rice rate, chalky grain rate, chalkiness degree, and length-width ratio, indicating that processing and appearance quality were maintained. In terms of eating quality, gel consistency was significantly increased, while amylose and protein contents were slightly reduced, suggesting a significant improvement in taste, supported by farmer sensory evaluation (**Table 3**). At the same time, evaluation by the Plant Protection Institute of Jiangsu Academy of Agricultural Sciences confirmed that the rice blast resistance level of Xinhuai 5 was higher than that of the control Huaidao 5 (**Table 3**).

**Table 3:** Rice quality evaluation and rice blast resistance evaluation.

Variety	Roughness rate /%	Precision of rice /%	Head rice rate/%	Grain length/mm	Grain length/width ratio	Chalky grain percentage/%	Chalkiness (Yin glutinous rice percentage) /%	Amylose content/ %	Gel consistency/mm	Protein content/%	Aggregative index number		Resistance evaluation
											index	grade of disease	
**Huaidao 5**	85.3	76.0	74.3	5.2	1.8	54	12.8	16.0	58	7.43	5.00	5	MS
**Xinhuai 5**	84.3	75.3	73.4	5.2	1.8	56	13.3	15.8	66	7.41	4.25	5	MS

MS: the rice blast resistance category is moderately susceptible.

## 4 Discussion

### 4.1 Mechanism underlying improved NUE in Xinhuai 5

Improving NUE is essential for sustainable agriculture, reducing fertilizer input while maintaining yield. This study demonstrated that introgressing the *NRT1.1B* gene from *indica* into the *japonica* variety Huaidao 5 significantly enhanced NUE in the improved Xinhuai 5 line. Consistent with the improved phenotype, Xinhuai 5 exhibited significantly higher ¹^5^N accumulation in shoots and elevated expression of key nitrogen metabolism genes (*NRT1.1B, NRT2.1, NIA1, NIA2, NIR1, NIR2*), indicating enhanced nitrate uptake and assimilation capacity at the molecular level.

Dr. Hu introduced the *indica NRT1.1B* into *japonica* rice to improve the nitrogen use efficiency of *japonica* rice and obtain higher yield, which is of great value in improving the NUE and yield of *japonica* rice (Duan and Zhang, 2015; Hu et al., 2015). The chlorate resistance assay indicated that Xinhuai 5 was more sensitive to chlorate than the recurrent parent, further confirming its improved nitrogen uptake and utilization capacity. This was consistent with previous reports that chlorate sensitivity was correlated with enhanced nitrate assimilation (Xin et al., 2014; Hu et al., 2015). Similar improvements in NUE have been achieved through regulating key genes such as *OsNLP4* (Wu et al., 2021) and *OsNRT2.3b* (Fan et al., 2016), which promote nitrate transport and assimilation.

Nitrogen application experiments revealed that the improved Xinhuai 5 exhibited earlier flowering, reduced plant height, enhanced nitrogen use efficiency, and effective tillering, seed setting rate, and grain yield across varying nitrogen regimes compared to the control. These phenotypic improvements, including higher tiller number, shortened growth period, and improved lodging resistance, collectively enhance suitability for rice–wheat double cropping systems. These results aligned with previous findings that *NRT1.1B* and other NUE-related genes, such as *Hd10-1*, *OsAAP1*, *OsNRT2.3b*, *OsAAP3*, and *OsAAP1*, contributed to improve nitrogen assimilation and agronomic performance (Mekouar, 2018).Furthermore, the differential response of Xinhuai 5 to varying nitrogen doses provides further insights into the physiological basis of its improved NUE (**Figure 6**). Across all four nitrogen application levels (0, 150, 240, and 330 kg N hm^-^²), Xinhuai 5 consistently maintained a higher nitrogen harvest index and greater shoot nitrogen accumulation compared to Huaidao 5 (**Figures 6A, B**). This indicated that the enhanced NUE in Xinhuai 5 primarily from intrinsic improvements in nitrogen partitioning efficiency. While root ¹^5^N accumulation was comparable between the two lines, shoot ¹^5^N content was significantly higher in Xinhuai 5 (**Figure 7A**), further supporting enhanced nitrogen translocation capacity. This genotype-dependent nitrogen partitioning pattern is consistent with the previous study (Becheran and Corzo, 2024). Becheran et al. reported that nitrogen-responsive sunflower line (B481-6) exhibited enhanced nutrient translocation and yield under increased nitrogen availability, in contrast to the non-responsive line (R453). These findings indicated Xinhuai 5 exhibited intrinsic improvement in nitrogen mobilization, rather than soil degradation effects across diverse nitrogen regimes.

**Figure 8:**
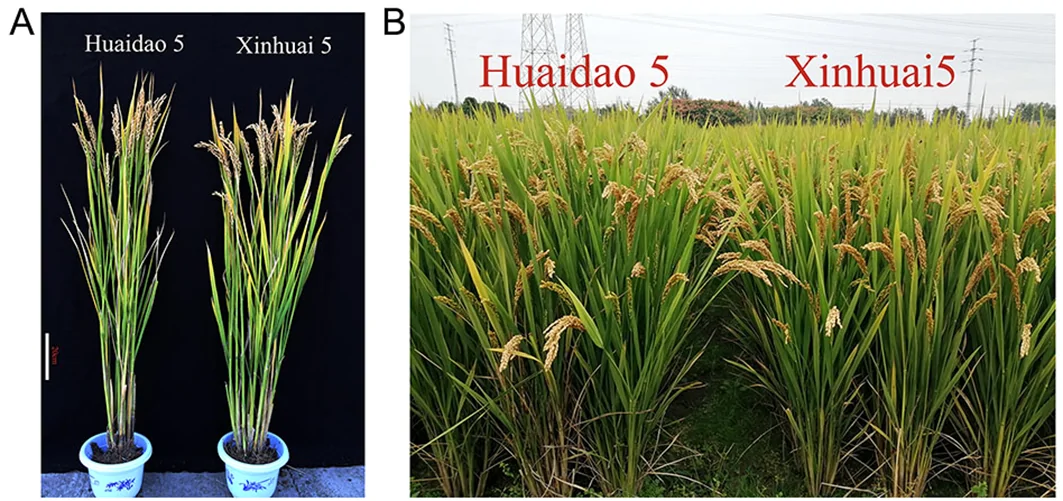
Performance of Xinhuai 5 and Huaidao 5. **(A)** Single plant performance of Xinhuai 5 and Huaidao 5, scale = 20 cm. **(B)** Field performance of Xinhuai 5 and Huaidao 5.

Notably, the field NUE test of Xinhuai 5 showed that in the direct seeding planting mode, the yield increase ratio of Xinhuai 5 under the nitrogen reduction treatment (5.89%) was significantly higher than that under the normal nitrogen application treatment (0.69%). Under a 30% nitrogen reduction, Xinhuai 5 achieved yields comparable to those in Huaidao 5 under full fertilization across both direct seeding and machine transplanting systems. Dr. Hu found that the yield of *japonica* rice varieties containing *NRT1.1B* increased by 30-33% under semi-fertilization conditions, while the NUE increased by 30%. Under normal nitrogen application conditions, the yield increased by 8-10%, and the NUE increased by about 10% (Hu et al., 2015). In addition, after years of multi-point planting observation, it was found that the plant height of the improved line Xinhuai 5 was reduced by 2–5 cm, the growth period was advanced by 3–5 days, and the tiller number was increased by 1–2 compared with that observed for the parent Huaidao 5. Additionally, Xinhuai 5 maintained the advantages of Huaidao 5, such as fast filling rate, high rice yield, good late maturity, and strong lodging resistance (**Figure 8**). When the total nitrogen content was reduced to about 77% (urea was reduced to 70%), the yield level of Xinhuai 5 was equivalent to that of Huaidao 5 under the normal fertilization level (300 kg/hm^2^), indicating that the NUE of Xinhuai 5 was higher. This is conducive to agricultural cost-saving and efficiency-increasing, farmers’ income increasing, and the implementation of eco-friendly rural policies.

Therefore, the mechanism of high nitrogen use efficiency of nitrogen-efficient rice was discussed when comparing the yield, NUE, and field performance under different nitrogen levels. This provided a basis for the breeding of nitrogen-efficient rice varieties and reliable germplasm and technical support for the promotion of nitrogen-efficient *japonica* rice varieties.

In addition to the enhanced NUE phenotype, our study also revealed a differential physiological response to potassium chlorate stress between Huaidao 5 and Xinhuai 5. Quantitatively, chlorate treatment reduced carotenoid content by 46.38% in Huaidao 5 and by 31.65% in Xinhuai 5, indicating that Huaidao 5 was more severely affected. Mechanistically, this differential response can be explained by the chemical properties of potassium chlorate and its physiological consequences. As a strong oxidant, chlorate induces excessive reactive oxygen species (ROS) accumulation upon absorption, leading to rapid consumption and degradation of carotenoids during antioxidant defense (Young, 1991; Havaux, 1998). Meanwhile, the structural similarity between ClO_3_^-^ and NO_3_^-^ causes competitive inhibition of nitrate reductase, disrupting nitrogen utilization and subsequent carotenoid biosynthesis (Nunes-Nesi et al., 2010). These oxidative and metabolic insults synergistically damage chloroplast membrane integrity, accelerating the release and decomposition of carotenoids, with eventual leaf chlorosis and desiccation (Cazzonelli, 2011). Carotenoid depletion further compromises photosynthesis and exacerbates photooxidative damage, and further impairs water regulation. The more severe reduction in Huaidao 5 likely reflects its poor antioxidant capacity and metabolic regulation compared with Xinhuai 5, which is consistent with the enhanced stress tolerance associated with the introgression of NRT1.1B from indica into the japonica background (Hu et al., 2015). Collectively, these results suggest that the improved NUE and stress tolerance in Xinhuai 5 may be attributed not only to enhanced nitrogen utilization but also to a more robust antioxidant system and metabolic regulation under adverse conditions.

### 4.2 Molecular marker-assisted breeding

Molecular marker-assisted selection technology offers an efficient strategy for precision breeding. Not affected by environmental factors, it can accelerate the breeding of new rice varieties with ENU (Hasan et al., 2021). In this study, a functional KASP marker was developed based on an SNP (C/T) within *NRT1.1B*, enabling accurate discrimination between *indica* and *japonica* alleles and efficient selection of positive progeny in backcross generations. This approach significantly shortened breeding time and improved selection accuracy, which was consistent with successes in marker-assisted introgression of other agronomic genes (Qi et al., 2022). The use of molecular marker-assisted selection also facilitated the recovery of the recurrent parent genome, minimizing linkage drag and preserving essential traits such as grain quality and blast resistance.

In addition, molecular marker-assisted breeding should consider the detection of the target gene and genetic background (Hittalmani et al., 2000; Gouda et al., 2013). In this study, *NRT1.1B-KASP* marker detection and multiple backcrosses were used during the backcross transfer of nitrogen-efficient genes to obtain lines containing the target gene. The results showed that the experimental scheme of this study is feasible. The donor and recipient parents were conventional varieties of *japonica* rice, and the genetic distance was relatively small. Homozygous lines were obtained after five directional backcrosses. Some scholars introduced the major QTL gene for cadmium uptake into the superior rice variety ‘Yueguang’ through backcross breeding. This could significantly reduce the accumulation of Cd in rice grown in cadmium-contaminated soil and provide an effective superior genetic resource for low-cadmium rice breeding (Yu et al., 2022).

In this study, the nitrogen efficiency gene *NRT1.1B* was introduced into the high-quality, high-yield, and late-maturing medium *japonica* variety Huaidao 5. The results showed that the NUE of the improved line was significantly better than that of the donor parent Huaidao 5. Through field experiments at four nitrogen application levels, it was shown that the nitrogen accumulation of Xinhuai 5 increased with the increase of nitrogen application, and the nitrogen harvest index and nitrogen use efficiency showed downward trends. The researchers conducted a two-year field plot and micro-plot experiment by controlling nitrogen application rate and planting density to study the effects of reduced nitrogen and increased density cultivation on yield, nitrogen fertilizer fate, and root characteristics of double-cropping rice. The results showed that increasing density cultivation under the condition of reducing nitrogen application by 20% could maintain high yield of rice and significantly improve the agronomic efficiency of nitrogen fertilizer (Hu et al., 2024). The whole growth period of improved Xinhuai 5 was significantly shorter than that of Huaidao 5, and the yield was significantly increased. Some scholars have found that the key gene *OsDREB1C*, controlling nitrogen absorption in rice, can enhance the ability of rice to absorb and transport nitrogen, allocate more nitrogen to grains, and increase nitrogen use efficiency by 25.8-56.6% compared with that in the control group (Wei et al., 2022). The improved crops bloom faster, which means that the planting period is shortened and the production efficiency is improved. In today ‘s increasingly severe climate change period, it also means that rice production can be completed before the high temperature in summer (Wei et al., 2022). In addition, the results of rice quality evaluation and rice blast resistance identification of Xinhuai 5 and Huadao 5 showed that there was no significant difference in processing and appearance quality. The eating quality and rice blast resistance level of Xinhuai 5 were superior to those of Huaidao 5.

Therefore, in the future breeding of high nitrogen use efficiency varieties, we should pay attention to the combination of molecular marker selection and conventional breeding methods; consider the resistance of important pests, diseases, and rice quality; and strive to improve the resistance, yield, and quality simultaneously.

## 5 Conclusion

In this study, we successfully developed a high-throughput *NRT1.1B-KASP* functional marker and applied it in molecular marker-assisted selection to introduce the *indica*-type *NRT1.1B* allele into the elite *japonica* variety Huaidao 5. The improved line, Xinhuai 5, exhibited significantly enhanced NUE, achieving yields equivalent to the recurrent parent under 30% less nitrogen fertilizer, accompanied with improved agronomic traits such as reduced plant height, earlier maturity, and increased tillering. Importantly, grain quality and blast resistance were maintained or improved. These findings demonstrated that functional marker-assisted selection of *NRT1.1B* was a highly effective strategy for developing nitrogen-efficient *japonica* rice varieties. Our findings support sustainable agriculture by reducing fertilizer use and environmental impact without compromising yield or quality.

## Data Availability

The original contributions presented in the study are included in the article/**Supplementary Material**. Further inquiries can be directed to the corresponding authors.
