# Supplementary material for: Improving nitrogen use efficiency of japonica rice by molecular marker-assisted selection of the NRT1.1B gene
Source: Front Plant Sci. 2026 Aug 17;17:1786744. doi: 10.3389/fpls.2026.1786744 (PMC13539727; doi:10.3389/fpls.2026.1786744)
Supplement: Supplementary file 1 [file SupplementaryFile1.docx]

**Supplementary Figure 1 |** KClO_3_ resistance of Xinhuai 5 and Huaidao 5 (CR %). KClO_3_ resistance (CR%) = (Average seedling length in KClO_3_) / Average seedling length in water × 100%
